# Expression of Phospholipase D Family Member 6 in Bovine Testes and Its Molecular Characteristics

**DOI:** 10.3390/ijms241512172

**Published:** 2023-07-29

**Authors:** Rui Yang, Boyang Zhang, Wenqian Zhu, Chunling Zhu, Lanxin Chen, Yansen Zhao, Yueqi Wang, Yan Zhang, Amjad Riaz, Bo Tang, Xueming Zhang

**Affiliations:** 1 State Key Laboratory for Zoonotic Diseases, College of Veterinary Medicine, Jilin University, Changchun 130062, China; ruiyang22@mails.jlu.edu.cn (R.Y.); zhangboyang199807@163.com (B.Z.); zhuwq21@mails.jlu.edu.cn (W.Z.); zcl18937379639@163.com (C.Z.); tang_bo@jlu.edu.cn (B.T.); 2 Department of Theriogenolog and University of Veterinary and Animal Sciences, Lahore 54000, Pakistan; dramjadriaz@uvas.edu.pk

**Keywords:** bovine, bioinformatics, phospholipase D family member 6, testes, spermatogonial stem cells

## Abstract

Spermatogonial stem cells (SSCs) are the only primitive spermatogonial cells in males that can naturally transmit genetic information to their offspring and replicate throughout their lives. Phospholipase D family member 6 (PLD6) has recently been found to be a surface marker for SSCs in mice and boars; however, it has not been validated in cattle. The results of reversed transcription-polymerase chain reaction (RT-PCR) and quantitative real-time PCR (qRT-PCR) found that the relative expression of the *PLD6* gene in the testicular tissues of two-year-old Simmental calves was significantly higher than that of six-month-old calves. Immunofluorescent staining further verified the expression of PLD6 protein in bovine spermatogenic cells like germ cell marker DEAD box helicase 4 (DDX4, also known as VASA). Based on multiple bioinformatic databases, PLD6 is a conservative protein which has high homology with mouse Q5SWZ9 protein. It is closely involved in the normal functioning of the reproductive system. Molecular dynamics simulation analyzed the binding of PLD6 as a phospholipase to cardiolipin (CL), and the PLD6-CL complex showed high stability. The protein interaction network analysis showed that there is a significant relationship between PLD6 and piwi-interacting RNA (piRNA) binding protein. PLD6 acts as an endonuclease and participates in piRNA production. In addition, PLD6 in bovine and mouse testes has a similar expression pattern with the spermatogonium-related genes *VASA* and piwi like RNA-mediated gene silencing 2 (PIWIL2). In conclusion, these analyses imply that PLD6 has a relatively high expression in bovine testes and could be used as a biomarker for spermatogenic cells including SSCs.

## 1. Introduction

Phospholipase D family member 6 (PLD6) is the sixth member of the phospholipase superfamily, known as Zucchini (also known as MitoPLD) in Drosophila. The main function of PLD6 appears to be the production of signaling lipid phosphatidic acid (PA) on the mitochondrial surface, which is involved in the regulation of mitochondrial division and fusion, and thus functions as a phospholipase [1,2]. The function and dynamics of mitochondria are closely related to multiple biological processes such as cell growth, proliferation and differentiation [3,4]. Additionally, PLD6 is also involved in the production of primary piwi-interacting RNA (piRNA) which regulates male germ cell development and genome stability. Whereas in the primary piRNA biogenesis, PLD6 functions as a nucleic acid endonuclease [5,6,7]. In male mice deprived of PLD6, the quantity of piRNAs during spermatogenesis is drastically reduced, meiosis is blocked, and ultimately the main phenotype is sterility [8,9]. This evidence suggests that PLD6 plays an essential role in the development of male germ cells.

Spermatogenesis is a complex physiological process that starts with the differentiation of spermatogonia and ends with the formation of mature sperm. Spermatogonia reside in the base of the seminiferous epithelium and are generally divided into three categories, type A, intermediate and type B spermatogonia. For most animals, a small population of type A spermatogonia are regarded as spermatogonial stem cells (SSCs). As the only primitive germ cells in males, SSCs transmit genetic information to their offspring and replicate throughout their lives by self-renewal, proliferation and differentiation. They play an irreplaceable role in the spermatogenesis and germline evolution [2,10]. It is generally believed that SSCs reside in the microenvironments/niches of the seminiferous tubules. These niches consist of Sertoli cells, Leydig cells, the basement membrane and peritubular myoid cells, providing SSCs with a microenvironment for growth and growth factors needed to maintain their plasticity [11]. Although they are the foundation of continuous spermatogenesis, SSCs have relatively low numbers in the testis, approximately accounting for only 0.02–0.03% of the cell suspension of the whole testis [12]. Thus, identification of these cells by varied methods including biomarker validation is essential to elucidate their functions. At present, surface labeling of SSCs has made significant progress in rodents [13,14]. In domestic animals, some markers such as GDNF family receptor alpha-1 (GFRα-1), Promyelocytic leukaemia zinc finger (PLZF) [15], Thy-1 Membrane Glycoprotein (THY1), and Ubiquitin C-terminal hydrolase 1 (UCHL1) [16] have been used to enrich and identify the undifferentiated spermatogonia. Recent studies have shown that PLD6 is expressed in the plasma membrane of mouse SSCs [17] and can be a molecular marker for pre-sexually mature porcine SSCs [18]. However, it has not been confirmed yet in bovine undifferentiating spermatogonia including SSCs.

In this study, reverse transcriptase polymerase chain reaction (RT-PCR), quantitative real-time PCR (qRT-PCR) and immunostaining were used to reveal the expression and localization of PLD6 in bovine testes. The bioinformatics techniques were further employed to investigate the secondary structure and molecular characteristics, as well as possible functions and signaling pathways of PLD6 in bovine spermatogenesis.

## 2. Results

### 2.1. Expression of PLD6 in Bovine Testes

**Figure 1:**
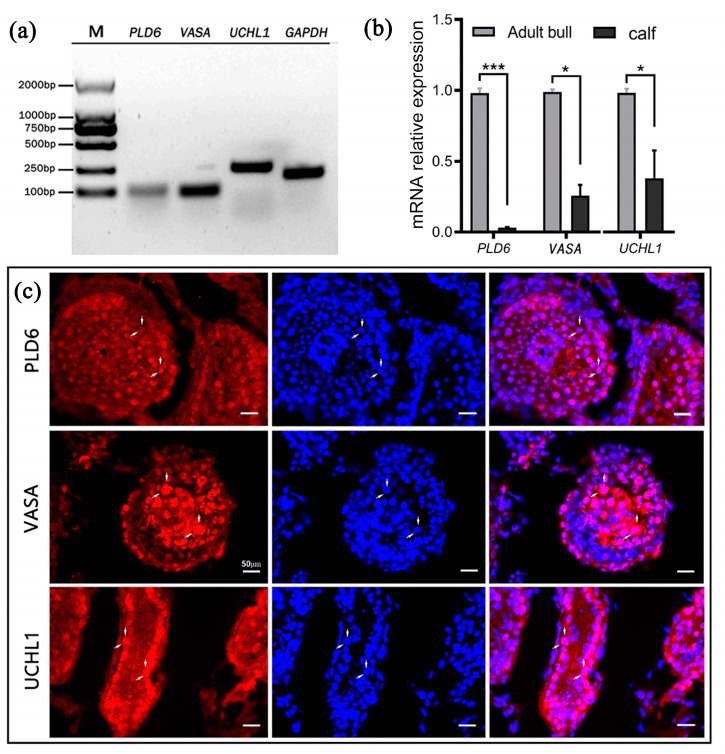
PLD6 expression in bovine testes: (**a**) Electrophoresis of RT-PCR products (*PLD6*, *VASA*, *UCHL1*); (**b**) mRNA relative levels of *PLD6*, *VASA*, *UCHL1* by qRT-PCR analysis (* *p* < 0.05, *** *p* < 0.001); (**c**) Immunofluorescent staining of PLD6, VASA and UCHL1 (red: PLD6, VASA and UCHL1 positive staining; blue: DAPI counterstained nuclei; purple: merged images; white arrows refer to spermatogonial stem cells). Scale bars = 50 μm.

The RT-PCR and qRT-PCR analyses revealed that, with *GAPDH* as the internal reference, *PLD6*, *UCHL1* and the germ cell marker *VASA* all transcriptionally expressed in both 6-month-old and 2-year-old bovine testes (Figure 1a,b). Immunofluorescent staining showed that PLD6 was observed in all germ cells similar to VASA in the seminiferous epithelium, while UCHL1 was mainly detected in spermatogonia on the basement membrane of the seminiferous tubules (Figure 1c).

### 2.2. Phylogenetic and Structural Features of Bovine PLD6

**Figure 2:**
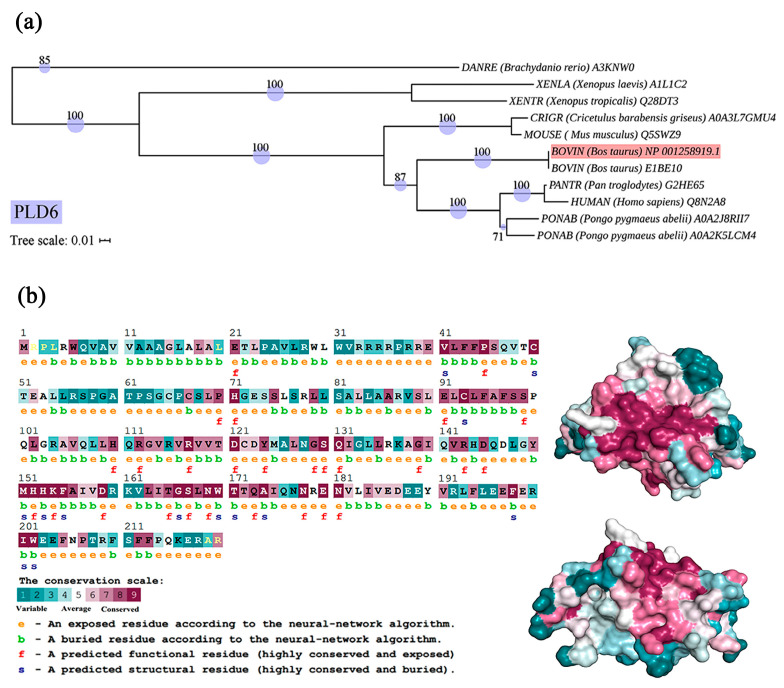
Phylogenetic and structure analysis of PLD6: (**a**) Evolutionary Trace Server was used for PLD6 phylogenetics. Single Likelihood Ancestor Counting algorithm module of Datamonkey Adaptive Evolution Server was employed to analyze the evolutionary pressure. ClustalX 1.83, MEGA7.0 and iTOL were used to construct and visualize the phylogenetic tree. (Bar: Nucleotide divergence, numbers at the nodes indicate the bootstrap values); (**b**) Protein structure analysis of PLD6 including homology modeling and conservative analysis by SWISS-MODEL & convolutional neural network of Consurf Web Server (left), and structure prediction by the Predict Protein (right, showing two opposite sides); e: an exposed amino acid residue; b: a buried residue with low solvent accessibility; f: a highly conserved and exposed residue; s: a highly conserved and buried residue.

The phylogenetic tree was constructed by Neighbour-Joining, all the bootstrap values were >70, indicating that the phylogenetic tree has high credibility. It demonstrated that the PLD6 protein of our study grouped together with bovine BIBE10 protein of the same genus, indicating a similar degree of evolution. Additionally, bovine PLD6 exhibited a close relation to mouse Q5SWZ9 protein, with a bootstrap value of 100. The length of the branch in the tree was correlated to the level of genetic variability, indicating that bovine PLD6 is more evolved than that in mice, but less evolved than that in Homo sapiens (Figure 2a). Evolutionary trace analysis (Table 1) showed that PLD6 contained 34 important trace residues that are subject to evolutionary pressure (Importance score < 25%), in which 17 residues are subject to the most prominent selection pressure (Score < 5%). The Single Likelihood Ancestor Counting (SLAC) algorithm revealed that the positively selected sites account for 19.33%, while the purification selection accounts for 30.57%. The detailed information on the species involved in the phylogenetic tree is shown in Table 2.

As shown in Figure 2b, homology modeling and conservation analysis exhibited that PLD6 protein has a “bell-shaped” structure, including 37.72% α-helix, 9.25% β-sheet, 43.64% random coil and other secondary structures. The neural convolutional network predicts the solvent accessibility of amino acid residues, showing that 41.36% of PLD6 amino acids have low solvent accessibility and are buried (marked “b”), while 58.63% of PLD6 amino acids are exposed (marked “e”). The structural and functional properties of conserved residues were analyzed by the convolutional neural network (CNN) of the Consurf server. Among them, residues that are highly conserved and exposed on the protein surface (functional residue, marked “f”), are thought to be involved in the biological functions of PLD6, and residues that are highly conserved and buried inside the protein (structural residue, marked “s”), are important for the maintenance of the structural features of the protein. This finding well constructs the correlation between protein structural features and function. Most of the conservative amino acids are located on the surface, which is speculated to be involved in the functioning of PLD6. Like most PLD family members, PLD6 has several highly conserved regions (Figure 2b), including Val41-Ser46 (VLFFPS), Glu91-Ser99 (ELCLFAFSS), Met151-Ala156 (MHHKFA) and Leu163-Trp170 (LITGSLNW). The prediction of subcellular location by the PSORT Prediction server shows that PLD6 is located in the outer mitochondrial membrane, and the Val10-Val32 sequence is a transmembrane segment by TMHMM 2.0 analysis.

### 2.3. Molecular Dynamics Simulation and Binding Mode Analysis

**Figure 3:**
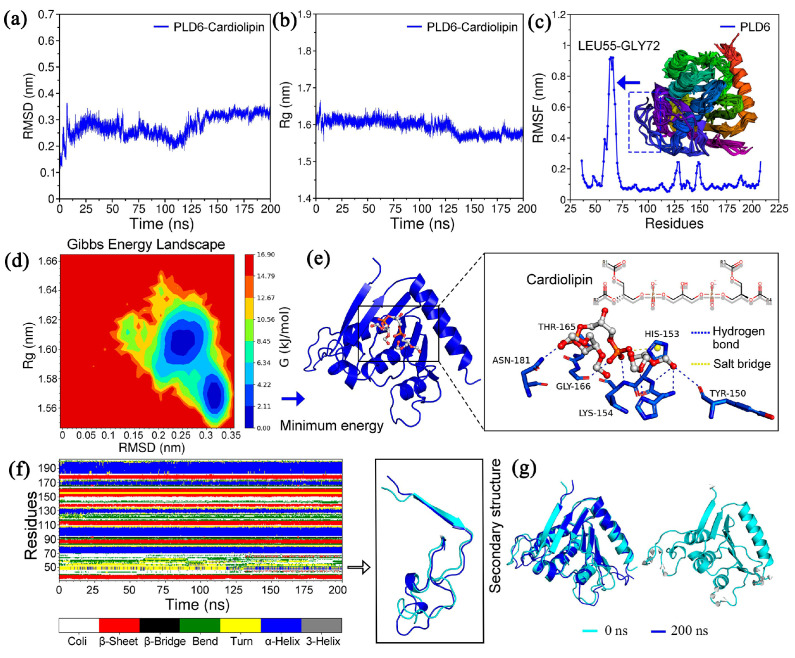
Molecular dynamics simulation of the PLD6-CL complex: (**a**) RMSD plot of PLD6-CL complex; (**b**) Rg plot of PLD6-CL complex; (**c**) RMSF and structural overlap plot of PLD6; (**d**) PLD6-CL complex free energy landscape (FEL); (**e**) Binding conformation of CL to residues inside the phosphodiesterase domain of PLD6; (**f**) Allosteric diagram of the secondary structure of PLD6; (**g**) Conformational differences of PLD6 at 0–200 ns.

Molecular dynamics simulation and binding mode analysis were all implemented using Gromacs (https://manual.gromacs.org/, accessed on 23 March 2021), which has built-in multiple commands. The analysis shows that the complexes PLD6-CL exhibit high stability during the simulation with a mean Root-Mean-Square Deviation (RMSD) value of 0.275 ± 0.042 nm (max = 0.363 nm). It was noteworthy that the RMSD values of the system gradually decreased after 35 ns, with a more pronounced conformational shift at about 115 ns, and maintained a significant steady state after 150 ns until 200 ns (Figure 3a). The radius of gyration (Rg) reflects the effect of CL molecules on the tightness of PLD6 folding, with values ranging from 1.548–1.667 nm (Figure 3b). Moreover, it was not difficult to find that the above structures have a high degree of overlap, i.e., when superimposing and comparing the PLD6 structures at 0–200 ns, that was, the binding of CL molecule did not significantly affect the PLD6 conformation, while increased the folding tightness and orderliness (Figure 3g). Meanwhile, the root means square fluctuation (RMSF) values of PLD6 in the complex were in the range of 0.052–0.921 nm, as shown in Figure 3c, and PLD6 contains a region with prominent structural flexibility in one segment (Leu55-Gly72). Overlapping the conformations at different moments, it was also found that the above region showed obvious structural changes, but the region that plays the function of phospholipase has high stability, which is favorable for the binding of CL molecule and hydrolysis reaction.

In addition, the relationship between protein Gibbs free energy and RMSD and Rg were revealed by using the principal component analysis (PCA) method, so as to clarify the correlation between relative free energy and protein conformation. The red region was the energy peak, the transition region fading from red to blue was the energy mound, which can be considered as the energy potential connecting the sub-stable states, and the dark blue part served as the energy lowland, indicating the aggregation region of the minimum conformation of relative Gibbs free energy (Figure 3d). Subsequently, the minimum energy confirmation was extracted and the binding mode analysis was performed. From Figure 3e, it could be seen that the O atoms abundant in the end groups of CL molecule could bind to residues Tyr150, His152, His153, Lys154, Thr165, Gly166 and Asn181 of the functional region of PLD6 phospholipase and form 9 hydrogen bonds. It was worth noting that the formed hydrogen bonds are symmetrically distributed around the CL molecule, and this binding pattern makes the force more uniform. During the simulation, it was also found that PLD6 forms about 7–10 hydrogen bonds with CL stably. Meanwhile, the aromatic ring of cationic residue His-153 forms a salt bridge with the P atom in the CL molecule, which is conducive to the stable fitting of the CL molecule with certain structural flexibility in this region and provides a structural basis for the subsequent hydrolysis reaction. In addition, consistent with the findings in RMSF, frequent transitions between secondary structures such as the turn and bend were also evident in the Leu55-Gly72 region (Figure 3f).

### 2.4. Protein-Protein Interaction (PPI) Network

**Figure 4:**
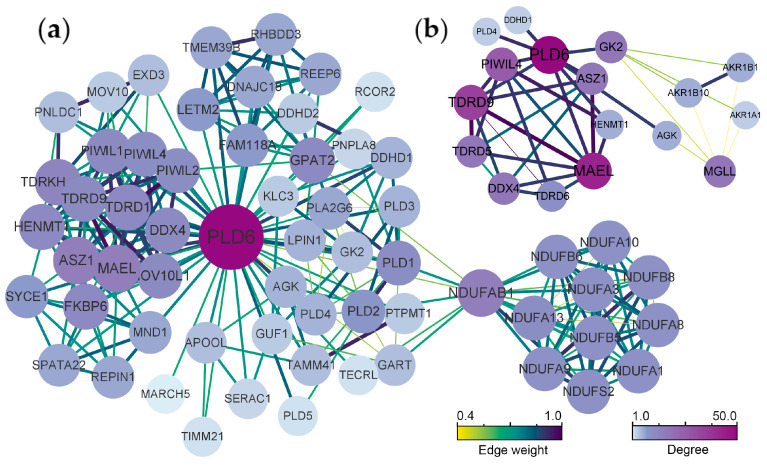
Protein-protein interaction (PPI) network: (**a**) Analysis of PLD6 protein interaction network. Protein interaction network analysis is enriched to 60 protein, which are divided into 5 main sub-networks by algorithm clustering; (**b**) The top 10 hub protein involving PLD6 were screened out, namely PIWIL4, TDRD9, MEAL, ASZ1, VASA (DDX4), GK2, MGLL, TDRD5, TDRD6 and HENMT1. Note: The color is assigned according to the degree of connection in the figure. The darker the color, the more critical the site is in the interaction network; the thicker the connection, the stronger the interaction between the two proteins.

The final average clustering coefficient (0.808) was obtained from the constructed PPI network, and the PPI enrichment *p*-value was <1.0 × 10^−16^. A total of 60 related proteins were enriched, which were clustered into 5 main sub-networks (Figure 4a). The top 10 hub protein including piwi like RNA-mediated gene silencing 4 (PIWIL4), tudor domain containing 9 (TDRD9), maelstrom spermatogenic transposon silencer (MAEL), ankyrin repeat, SAM and basic leucine zipper domain containing 1 (ASZ1), VASA (DDX4), lycerol kinase 2 (GK2), monoglyceride lipase (MGLL), tudor domain containing 5 (TDRD5), tudor domain containing 6 (TDRD6) and HEN methyltransferase 1 (HENMT1) were screened (Figure 4b). Obviously, PLD6 has a significant interaction with the above-mentioned protein. It is well known that protein with strong associations is often related to functions. Most of these proteins are related to the silencing of PIWI/piRNA pathway genes with promoter hypermethylation, indicating that PLD6 is mainly involved in piRNA metabolism.

### 2.5. Gene Ontology (GO) and Kyoto Encyclopedia of Genes and Genomes (KEGG) Analysis

**Figure 5:**
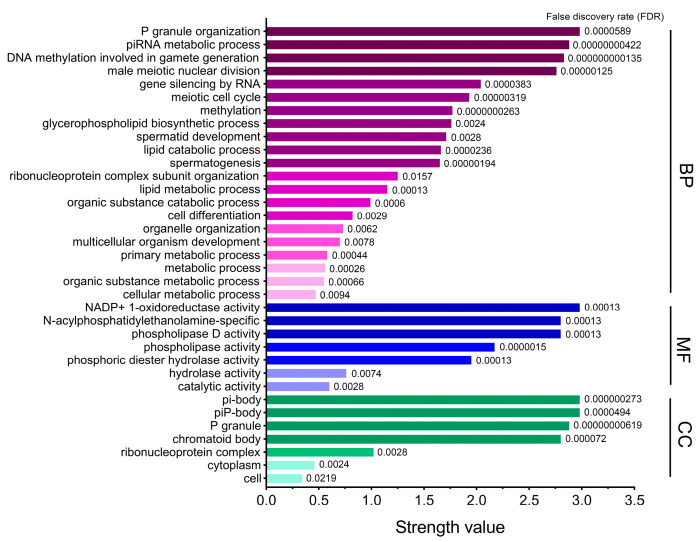
GO enrichment analysis of PLD6. The biological function of PLD6 is annotated from the three aspects of biological process (BP), molecular function (MF) and cell composition (CC). The greater the strength value, the higher the participation of PLD6 protein in this part.

As shown in Figure 5, GO analysis was used to annotate PLD6 from three aspects, biological process (BP), molecular function (MF), and cell composition (CC). The BPs of PLD6 are mainly the formation of P granule organization, piRNA metabolism, DNA methylation and male meiosis during the gamete formation. The formation of P granule organization promotes the production of polar granules in primordial germ cells of many organisms, while piRNA regulates the growth and development of germ cells and stem cells [19,20]. Additionally, PLD6 can promote DNA methylation during gametogenesis and facilitate the establishment of DNA methylation patterns in gametes [21]. The MF of PLD6 mainly involves phospholipase activity and participates in the catalytic hydrolysis of phosphodiester. For CC, PLD6 is mainly enriched in multiple membrane structures such as the outer mitochondrial membrane, outer cell membrane, and endoplasmic reticulum.

The regulation and metabolic pathways involving PLD6 were sorted from the KEGG database (Table 3). PLD6 is mainly involved in the metabolism of glycerophospholipid, aminophosphonate, amino acid, and ether lipid as well as the gonadotropin-releasing hormone (GnRH) signaling pathway in cattle. The participation rate of PLD6 in the glycerophospholipid metabolism pathway is 61.08%. As a phospholipase, PLD6 can hydrolyze CL on the outer mitochondrial membrane to produce phosphatidic acid, and participates in the regulation of mitochondrial division and fusion [5].

### 2.6. Multi-Sequence Alignment and Expression Analysis of PLD6 by GEO Mining

**Figure 6:**
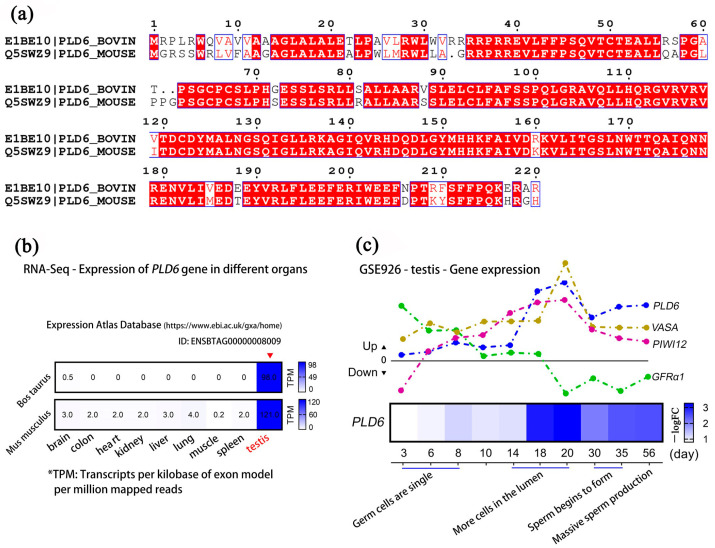
Dynamic analysis of PLD6/PLD6 expression level: (**a**) Bovine and murine amino acid multiple sequence alignment of PLD6 protein; (**b**) RNA-Seq-Expression of PLD6 in bovine and mouse organs including brain, colon, heart, kidney, liver, lung, muscle, spleen and testis, in which testis tissue is the most prominent; (**c**) Expression of PLD6, VASA, PIWIL2 and GFRα1 in mouse testes at 3, 6, 8, 10, 14, 18, 20, 30, 35 and 56 d. The upwards of the 0-tick mark means that the gene transcription level is up-regulated, and the downwards of the 0 scale means that the gene transcription level is down-regulated. The heat map indicates the transcription level of PLD6 at different time points.

Multi-sequence alignment (Figure 6a) shows that the amino acid sequence of bovine PLD6 has a high similarity with that of mice (Mean identity: 83.33%, Mean similarity: 94.59%). The sequences of Arg35-Arg218 regions are almost identical except for a few amino acids. The functional site of PLD6 playing a catalytic role is also the same. The GSE41637 data set in the GEO database and the ENSBTAG8009 entry in the EMBL-EBI database contain the transcriptomics sequencing data of various tissues of cattle and mice. They were employed for tissue-specific analysis of *PLD6*, showing its expression levels both in bovine testes and mouse testes are the highest, approximately 30–196 times compared with those in other tissues (Figure 6b). This suggests that *PLD6* is specifically expressed in bovine and mouse testicular tissues. The GSE926 data set records the relative expression of genes in mouse testes at 3, 6, 8, 10, 14, 18, 20, 30, 35, and 56 d (postnatal day). Among these genes, the expression of SSC-specific marker *GFRα1* shows a decreasing trend from 3 d to 20 d, then maintains a certain level afterward. The germ cell marker *VASA* and *PIWI12* show an up-regulated expression from 3 d to 56 d, with a peak at 18–20 d, then decreases to a certain level and remains. We noticed that the transcription of *PLD6* also shows a similar trend from 3 d to 56 d, indicating it has a similar expression pattern with *VASA* and *PIWI12* (Figure 6c).

## 3. Discussion

The wall of the seminiferous tubule is composed of multiple layers of cells, which can be divided into spermatogenic cells and sustentacular/Sertoli cells. Before sexual maturity, there are only spermatogonia in the seminiferous tubules except for immature Sertoli cells; after sexual maturity, there are spermatogonia, primary spermatocytes, secondary spermatocytes, spermatids and sperm from the base of the seminiferous tubules to the lumen [22]. The formation process from spermatogonia to sperm is spermatogenesis, which ultimately produces mature male gametes, and SSCs are the source of spermatogenesis. 

**Figure 7:**
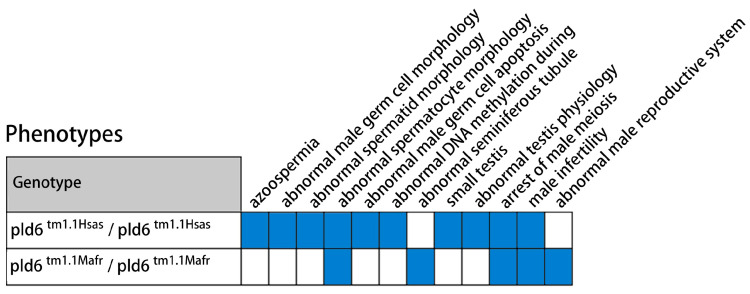
Phenotypic changes in *PLD6*^tm1.1Hsas^ and *PLD6*^tm1.1Mafr^ mice based on the references and MGI database.

Previous studies and the data mining in the mouse genome informatics (MGI) database (MGI, http://www.informatics.jax.org/, accessed on 20 April 2021) indicate that the absence of PLD6 significantly affects the development and function of the reproductive system (Figure 7). The phenotypic changes of *PLD6*^tm1.1Hsas^ and *PLD6*^tm1.1Mafr^ mutant mice are mainly abnormal sperm and seminiferous tubules, developmental disorders and dysfunction of the reproductive system [9]. Among them, azoospermia and the absence of epididymis are mainly manifested in seven-week-old mice. Physiological abnormalities of the testis are indicated by severe damage to the RNA interacting with PIWI. Abnormal sperm morphology is manifested by presenting atypical spermatocytes which have condensed or swollen nucleus in the seminiferous tubules. Small testes and arrested male meiosis are also seen in PLD6 mutants. In addition, studies have shown that PLD6 is expressed in the plasma membrane of mouse SSCs [17], and it has also been suggested as a molecular marker for SSCs in pre-sexual boars [18]. In this study, we utilized combined experimental verification and biometric analysis to investigate the potential role of PLD6 in bovine spermatogenesis.

Firstly, we verified the expression of *PLD6*/PLD6 in bovine testes both transcriptionally and immunohistochemically. Transcriptional analysis verified the expression of *PLD6* mRNA in bovine testis tissue, like undifferentiated spermatogonia marker *UCHL1* and germ cell marker *VASA* (Figure 1a,b). Immunostaining further indicates PLD6 expression in bovine spermatogenic cells like VASA (Figure 1c). Thus, it could be used as a potential molecular marker for SSCs in sexually immature bovine testis tissues like in porcine testis [18]. 

PLD6 is a conserved protein. Phylogenetic analysis revealed that bovine PLD6 had the highest homology with the homologous EIBE10, and the Q5SWZ9 protein from mice came next (Figure 2a). Evolutionary trace analysis assigned importance values to amino acid residues, with lower values indicating lower variability during the evolution of the residue and greater importance for protein structure and function. The PLD6 protein contained 34 important and evolutionarily conserved trace residues, among which 17 residues were under the most prominent selection pressure (Table 1). To further investigate and quantify the magnitude of evolutionary pressure, the SLAC method was used to infer the substitution rate of gene sites, and the Ka/Ks ratio was calculated to determine the ratio of non-synonymous rate (Ka) and synonymous rate (Ks). The smaller the Ka/Ks ratio, the lower the selection pressure and the more conserved the site. Positive selection sites account for 19.33%, and purifying selection sites account for 30.57%. We further predicted the secondary structure of PLD6 and analyzed the solvent accessibility of amino acid residues using a convolutional neural network. It was found that 41.36% of the PLD6 amino acid residues have low solvent accessibility and are located internally (labeled as “b”), while 58.63% of the residues are located on the surface (labeled as “e”). It should be noted that surface-exposed residues with high conservation (labeled as “f”) are closely related to the protein’s function, while conserved residues with high solvent accessibility (labeled as “s”) are important for maintaining specific structures [23] (Figure 2b). Like most members of the phospholipase D family, the PLD6 protein contains several highly conserved regions, and these regions are listed in the results. In addition, we predicted the subcellular localization of PLD6 and found that it is located in the outer mitochondrial membrane, which provides a reference for its biological function as a molecular marker. Up to now, similar structure elucidation studies were reported only for mouse PLD6 [5,7], however, no information is available for bovine PLD6. 

Based on the molecular characterization and structure-function analysis, we found that PLD6 has a conserved H(x)K(x4)D motif (characterizing an enzymatically active region), which is hypothesized to interact with CL to generate the signaling molecule PA during the development of round to elongated spermatozoa, thereby inducing mitochondrial fusion. Molecular dynamics simulations provide more high-resolution details on time scales, conformational changes and energies, which we used to analyze the binding of PLD6 as a phospholipase to CL. During the 200 ns simulation, the PLD6-CL complex exhibited high stability and binding to the CL molecule did not significantly affect the protein conformation of PLD6. In addition, PLD6 contains a segment with prominent structural flexibility, Leu55-Gly72, which is a Zinc ion-binding region, and a highly conserved and structurally stable amino acid in the Asp147-Ala174 region (a phospholipase active region). The above features are also beneficial for the phospholipid-catalyzed hydrolysis function. Notably, in the lowest energy conformation, seven residues, including Tyr150, His152, His153, and Lys154, etc. interact with the CL molecule in a hydrogen bond, and all of them are highly conserved (Figure 3). This finding suggests that there is a significant correlation between the structural features we elucidated and their biological functions, and these crucial residues could also be a key breakthrough in the structural study of PLD6 [7].

Studying protein interactions is beneficial in enhancing our comprehension of the connections between protein functions. The protein-protein interaction (PPI) network are composed of proteins that interact with each other through interactions that can be used to reveal life processes such as biological signaling, regulation of gene expression, energy and material metabolism, and cell cycle regulation. Proteins do not perform their functions alone, but work together with several proteins to accomplish biological events. The existence of interacting proteins is often functionally synergistic and coherent, and together they influence the biological processes, molecular functions and cellular components. Systematic analysis of protein interactions in biological systems provides a higher dimensional insight into how proteins work. In this study, we searched for a collection of proteins with PLD6 as the core through the PPI network, clarified the regulatory pathways involved in PLD6 from a global perspective, and analyzed their roles in spermatogenesis. The analysis indicates that PLD6 is closely related to the screened top 10 hub proteins, suggesting that they are also strongly functionally related. Studies have shown that male sterility is associated with promoter hypermethylation-related silencing of PIWI/piRNA pathway genes, including *ASZ1*, *HENMT1*, *DDX4*, *PLD6*, *MAEL*, *TDRD1* and *TDRD5* [24,25,26,27]. PIWIL14 is essential for ensuring piRNA maturation by participating in primary or secondary biogenesis pathways [28]. It is also reported that PLD6 is synergistically involved in the formation of sperm mitochondrial sheaths with glycerol kinase 2 [4]. 

Furthermore, GO analysis indicates that the cellular components of *PLD6* are mainly enriched in multiple membrane structures such as the outer mitochondrial membrane, the outer cell membrane and the endoplasmic reticulum. Studies reported that ectopic overexpression of PLD6 is localized in the outer mitochondrial membrane [29]. The endogenous PLD6 is localized in the plasma membrane of testicular germ cells [17,18], and is expressed in the Golgi apparatus of testicular germ cells, especially in sperm cells [30]. Subcellular fractionation of testicular cells showed that PLD6 is also expressed in other cellular components, such as cytoplasm, cell membrane, and nuclear components [9]. This evidence is consistent with our analysis and the immunostaining observations. 

Sperm needs to rearrange organelles such as mitochondria in the final stage of maturation. Abnormal mitochondria can cause male sterility, leading to oligospermia and asthenospermia [31]. However, the dynamic regulation mechanism of mitochondria during spermatogenesis is still unclear. The enrichment of the KEGG pathway in this study shows that *PLD6* is mainly involved in the metabolism of glycerophospholipids, and the participation rate is as high as 61.08%. As a phospholipase, PLD6 mainly hydrolyzes the CL on the outer mitochondrial membrane to produce PA, which in turn absorbs the phosphatase Lipin 1, converts PA into diglycerides and promotes mitochondrial division [2,8]. Additionally, PLD6 can continuously convert 1,2-diacylsn-glycerol into phosphatidylethanolamine and promote the formation of sperm mitochondrial sheaths to ensure the normal morphology of mitochondria and sperm tails [1,32]. PLD6 can also interact with oil kinase (Gyk)-like protein kinase 1 (Gykl1) and glycerol kinase 2 (Gk2) to induce the aggregation of PLD6 and PA-dependent mitochondria in cells. Both Gykl1 and Gk2 are specifically located in the mitochondria of sperm. Male mice lacking Gykl1 or Gk2 exhibit sperm tail defects, abnormal mitochondrial morphology, and disordered mitochondrial sheath formation [4]. *PLD6* overexpression promotes mitochondrial aggregation, while *PLD6* deletion results in the disappearance of mitochondrial aggregation and tends to make mitochondria split [1,8]. Therefore, PLD6 plays a lipase function in the testis by participating in the regulation of mitochondrial division and fusion. 

The GO analysis also revealed that the biological process of *PLD6* is mainly involved in the metabolic process of piwi-interacting RNA (piRNA). The piRNA is a type of small RNA with a length of about 30 nt isolated from mammalian germ cells, and mainly exists in mammalian germ cells and stem cells. It maintains the integrity of the animal germline genome by silencing transposons [33]. PLD6 has been proven to be a backbone-non-specific, single strand-specific nuclease, and its effect on germ cell development can be traced back to its function in the piRNA pathway. It cleaves either RNA or DNA substrates with similar affinity. The production of 5′ phosphate and 3′ hydroxyl termini suggests that it can directly participate in the processing of primary piRNA transcripts [7]. In mouse germ cells, PLD6 plays a conserved role in the piRNA production pathway and links lipid metabolism signals on the mitochondrial membrane with small RNA biogenesis [8,34,35]. In addition, the male *PLD6*^−/−^ mice showed dramatically decreased piRNAs, damaged structure of the nucleoid body, blocked spermatogenesis at the pachytene stage, and infertility [8,9]. These reports strongly support the PLD6 related phenotype annotation in the present study. The above analysis and evidence imply that PLD6 might regulate bovine spermatogenesis through the piRNA pathway. 

Next, we used multi-sequence alignment to further explore the amino acid sequence of bovine PLD6, which has a high similarity with that of mouse PLD6, also indicating the similar functional site of PLD6 playing a catalytic role. Subsequently, we analyzed the tissue-specificity of PLD6 in cattle and mice by gene expression omnibus (GEO) deep mining, which showed a significant highest expression in bovine testes and mouse testes. In testes, the specific expression pattern of SSC molecular markers can reflect the differentiation state of germ cells [36]. Studies demonstrated that the seminiferous epithelium at early prepubertal stages in bovine consists of gonocytes (prospermatogonia), SSCs and immature Sertoli cells [15,32]. Therefore, there are relatively more germline stem cells in early prepubertal testis and they can be marked by VASA, PIWI12, UCHL1, PLZF, GFRα-1 [15,16,37,38], etc. In mice, SSCs first appeared at 16.5d (E16.5d) embryonic stage. Cell proliferation experiments found that the number of SSCs increased significantly from E16.5d to 2d after birth, and their proportion in the total number of germ cells also increased [11]. By GEO database mining, the present study found that the transcription level of *PLD6* is low in the embryonic stage, and slightly increases at E15.5–E16.5d, remarkably rises on the 14th day after birth, significantly increased and reached peak on the 18th–20th day after birth. This expression trend and pattern of *PLD6* is very similar to those of *VASA* and *PIWI12*. The above analysis indicates that PLD6 has testicular tissue specificity, and its expression pattern is consistent with that of germ cell marker genes. PLD6 reflects the differentiation of SSCs, and it could be a potential marker for bull germ cells including SSCs at certain developmental stages.

## 4. Materials and Methods

### 4.1. Animal Tissues

The animal tissues were from 6-month-old and 2-year-old healthy Simmental calves/bulls provided by Jilin Changchun Haoyue Islamic Meat Co., Ltd. (Changchun, China). The testicular tissue in each group was from three animals, and six testicular samples were collected from bilateral testicles. Briefly, the testicles were immediately transported to the laboratory from the slaughter house using an insulated container having ice, soaked in 75% alcohol for 10 min, then washed twice with phosphate buffer saline (PBS) containing 1% Penicillin-Streptomycin (Gibco, Grand Island, NY, USA). The epididymis and tunica albuginea were peeled off aseptically, the testes were cut into 1 cm^3^ pieces and put into freezing vials containing 1 mL freezing medium as reported previously [39]. Subsequently, the samples were stored in liquid nitrogen for later use. The study was permitted by the Jilin University Institutional Animal Care and Use Committee for the use of animals/tissues (SY201903002).

### 4.2. RT-PCR and qRT-PCR

The total RNAs were extracted from above frozen-thawed bovine testicular tissues according to the instructions of the Animal RNA Extraction Kit (Beyotime, Shanghai, China), and the cDNAs were obtained by using RNA Reverse Transcription Kit (TransGen Biotech, Beijing, China). The primers (Table 4) were designed according to the gene sequences in the NCBI database and synthesized by Sangon Biotech (Shanghai, China). Reversed transcription-polymerase chain reaction (RT-PCR) and quantitative real-time PCR (qRT-PCR) reaction systems of 20 μL were prepared and the reactions were carried out according to the manufacturer’s instructions (TransGen Biotech, Beijing, China). The experiments were repeated three times independently, and the relative gene expressions were calculated according to the formula 2^−△△CT^. Data were expressed as mean ± SEM.

### 4.3. Immunostaining

Paraffin sections (4 μm-thick) were prepared from the frozen-thawed testicular tissues. Briefly, the tissues were fixed with 4% paraformaldehyde in PBS for 24 h. Gradual dehydration was achieved by immersing in 75% ethanol for 4 h, 85% ethanol for 2 h, 90% ethanol for 2 h, 95% ethanol for 1 h, and 100% ethanol two times for 30 min each. After incubation with xylene (2 × 10 min), the tissues were immersed in a 1:1 mixture of paraffin: xylene for 30 min, in 100% paraffin twice (each for 1 h), at 56–60 °C. Subsequently, the tissue samples were solidified and sliced. The sections were deparaffinized, rehydrated, and further subjected to ethylene diamine tetraacetic acid (EDTA) antigen retrieval buffer (pH 8.0). Then the sections were blocked with 3% bovine serum albumin (*w*/*v*) for 30 min. Subsequently, they were incubated overnight at 4 °C with the following primary antibodies, mouse anti-UCHL1 (1:2000; Boster, Wuhan, China), rabbit anti-VASA (1:200; GeneTex, Shanghai, China) and rabbit anti-PLD6 (1:200; Abcam, Cambridge, UK). The next day, the sections were incubated with Alexa Fluor 594-conjugated goat anti-rabbit/mouse IgG (1:500; Proteintech, Rosemont, IL, USA) for 60 min. The sections were again incubated in EDTA antigen retrieval buffer and heated in a microwave oven to remove the primary and secondary antibodies that had bound to the tissues. To visualize the nuclei, the sections were counterstained with 4′, 6-diamidino-2-phenylindole (DAPI, Beyotim Biotechnology, Shanghai, China) and incubated at room temperature for 15 min. Finally, the sections were covered with anti-quenching tablets and observed under a Nikon 80i fluorescence microscope. The sections stained with isotype IgG served as the negative control.

### 4.4. Bioinformatics

#### 4.4.1. Phylogenetic Analysis

Evolutionary Trace Server (ETS, http://mordred.bioc.cam.ac.uk/~jiye/evoltrace/evoltrace.html, accessed on 5 May 2021) was used for PLD6 phylogenetics. Single Likelihood Ancestor Counting (SLAC) algorithm module of Datamonkey Adaptive Evolution Server (http://www.datamonkey.org/, accessed on 7 May 2021) was employed to analyze the evolutionary pressure of its coding genes. The non-synonymous rate (Ka), synonymous rate (Ks), and Ka/Ks ratio were calculated as previously described [40]. The Ka/Ks > 1 indicates the target gene is subject to positive selection, Ka/Ks = 1 represents neutral evolution, while Ka/Ks < 1 suggests the gene is subject to purify selection. Thus Ka/Ks ratio indicated the choice of gene locus. Additionally, the software ClustalX 1.83 [41] and MEGA7.0 [42] as well as iTOL (https://itol.embl.de/, accessed on 26 May 2021) were used to construct and visualize the phylogenetic tree.

#### 4.4.2. Protein Structure Analysis

The Predict Protein (http://www.predictprotein.org/, accessed on 16 June 2021) was used to analyze the secondary structure of PLD protein. The SWISS-MODEL (http://swissmodel.expasy.org/, accessed on 17 June 2021) and Consurf Web Server (http://consurf.tau.ac.il/, accessed on 22 June 2021) were employed to perform the homology modeling and conservative analysis [43]. The conservation and solvent accessibility of the amino acids were mapped to the protein structure. The relationship between the solvent accessibility of PLD6 amino acid residues and its structure, as well as function, was predicted based on the neural convolutional networks. The key amino acids were further annotated with visualization software PyMOL. The PSORT Prediction (http://psort1.hgc.jp/form.html, accessed on 30 June 2021) was used to predict the subcellular localization of PLD6, and to analyze its related biological behaviors as molecular markers [44]. The transmembrane region prediction was performed with TMHMM 2.0 (https://services.healthtech.dtu.dk/services/TMHMM-2.0/, accessed on 1 August 2021).

#### 4.4.3. Molecular Docking

Utilizing PLD6 as the receptor protein, the three-dimensional (3D) structure of the bovine-derived PLD6 was obtained by performing homology modeling with SWISS-MODEL Server (https://swissmodel.expasy.org/, accessed on 7 August 2021). The structure file of cardiolipin (CL) was downloaded from the PubChem (https://pubchem.ncbi.nlm.nih.gov/, accessed on 13 August 2021) database, energy minimization based on the MMFF94 force field was performed and structure files were generated that could be used for docking. Subsequently, the AutoDock Tools 1.5.6 program was used to remove redundant solvent molecules, balance the charge and add hydrogen atoms for the receptor protein PLD6. At the same time, the center coordinates of the docking and the Grid box were set. Semi-flexible molecular docking of CL molecule to PLD6 was performed with the help of the AutoDock Vina program [45]. The final results that consider scoring and conformational fit were selected and visualized using the PyMOL v2.4.0 program [46].

#### 4.4.4. Molecular Dynamics Simulation

The all-atom molecular dynamics simulation program GROMACS 2022.03 [47] was used to perform all-atom molecular dynamics simulations of the docked complex of PLD6 and CL as the initial conformation, with a view to analyzing the mechanism of action present and verifying the reliability of the binding mode. Simulations of the complexes were carried out based on the Amber 99SB-ILDN force field, using the Antechamber [48] and Acpype [49] programs to generate topology files of the ligand molecules. A TIP3P water model and a dodecahedral solvation box were chosen and the system boundary was set at a closest distance to the protein of 1.5 nm. One Na^+^ was randomly added to neutralize the system charge, and the energy of the system was minimized using a maximum of 50,000 steps of the fastest descent algorithm. The system was then ramped up to 310 K in a 500 ps simulation under a regular ensemble (NVT) and continued for 500 ps under an isothermal isobaric ensemble (NPT). After the system reached equilibrium, the bond lengths and bonding interactions were constrained by the LINCS algorithm, with step intervals set to 2 fs and 200 ns of unconstrained dynamic simulations. The trajectory of the calibration periodicity was analyzed by the GROMACS tools, in which the root mean square deviation (RMSD) indicates the degree of variation in molecular structure to measure the stability of the complex system; the root mean square fluctuation (RMSF) shows the structural flexibility of the protein and the fluctuation of residues; the radius of gyration (Rg) reflects the tightness of the protein folding during the simulation; The clustering results of the lowest Gibbs free energy were presented by free energy morphology and the lowest energy conformation was captured to analyze the binding mode of CL molecule to PLD6 protein.

#### 4.4.5. Protein-Protein Interaction (PPI) Network

The PPI network involving PLD6 was analyzed and visualized by the STRING (https://string-db.org, accessed on 15 August 2021) and Metascape databases. The STRING default medium confidence score ≥ 0.4, high confidence score ≥ 0.7, and highest confidence score ≥ 0.9 were used to create the PPI network. Notably, the mean confidence score of the proteins interacting with PLD6 was 0.736, which reached the high confidence level of the STRING database, indicating that the PLD6-based PPI network is able to enrich the truly relevant proteins. The Max number of interactors (both the 1st and 2nd shells) was set to ≤ 50, and the Markov Clustering algorithm (MCL) was used to cluster the proteins with close functions, so as to obtain the key proteins and sub-networks in the network. Subsequently, the Cytoscape plug-in Cytohubba was used to screen the proteins with the degree of connectivity at the top 10 in the protein network as hub proteins (highly connected proteins).

#### 4.4.6. GO and KEGG Analysis

The DAVID (https://david.ncifcrf.gov/, accessed on 20 August 2021), Uniprot (https://www.uniprot.org/, accessed on 23 August 2021), Metascape (https://metascape.org/, accessed on 27 August 2021), and GSEA (Gene Set Enrichment Analysis) databases were used to search and enrich the function as well as biological significance of *PLD6* at the Gene Ontology (GO) level. The pathways involving *PLD6* were associated and analyzed through the Kyoto Encyclopedia of Genes and Genomes (KEGG, https://www.kegg.jp/, accessed on 30 August 2021) database and the TBtools program.

#### 4.4.7. Multiple Sequence Alignment and GEO Database Mining

The sequence files were obtained from the NCBI and UniProt databases, and aligned using the Clustal Omega program. The alignment results were imported into the ESPript3 Server (https://espript.ibcp.fr/ESPript/cgi-bin/ESPript.cgi, accessed on 1 September 2021) and annotated. The transcriptomics sequencing (RNA-Seq) data of testicular tissues in GEO database (https://www.ncbi.nlm.nih.gov/geo/, accessed on 3 September 2021) were searched. The GEO2R and R languages were used to process the sequencing data. Taking |logFC| > 1.5 and *p* < 0.05 as the significant standard, the differentially expressed genes (DEGs) in each data set were screened. Finally, the expression patterns of *PLD6* in testicular tissues of cattle and mice in the RNA-Seq data were analyzed by using the Expression Atlas online platform (https://www.ebi.ac.uk/gxa/home, accessed on 7 September 2021) in the EMBL-EBI database (https://elixir-europe.org/, accessed on 10 September 2021).

### 4.5. Statistical Analysis

Statistical significance was compared among the groups. SPSS 22.0 and GraphPad Prism 8.3.0 software were used for data statistics and graphing. *t*-test was used for significance analysis of the univariate experiment. * *p* < 0.05, ** *p* < 0.01 and *** *p* < 0.001 indicate there is a statistical difference, or the difference is extremely significant.

## 5. Conclusions

In conclusion, we verified the expression of *PLD6*/PLD6 in bovine testis, analyzed its molecular features and predicted its possible functions in spermatogenesis by multiple bioinformatics. On the one hand, PLD6 might combine with cardiolipin as a phospholipase to induce mitochondrial fusion; on the other hand, PLD6 might participate in the processing of primary piRNA transcripts as an endonuclease and have a significant relationship with piRNA binding protein. We have found that, overall, PLD6 has a relatively high expression in bovine testes and could be used as a biomarker for spermatogenic cells including SSCs. Nevertheless, more and direct methods such as RNA interference and gene editing might be used to further elucidate the function of bovine PLD6 in the future.

## Figures and Tables

**Table 1:** The residues in PLD6 classified by importance and evolutionary pressure.

Importance Score	Number	Trace Residues
<25%	34	44, 45, 69, 71, 72, 83, 86, 93, 95, 97, 106, 109, 112, 113, 114, 116, 121, 151, 152, 154, 155, 159, 166, 167, 169, 170, 171, 174, 178, 180, 181, 194, 198, 202
<5%	17	86, 93, 95, 112, 121, 152, 154, 159, 166, 167, 169, 170, 174, 178, 180, 198, 202

**Table 2:** Species information in phylogenetic analysis.

Mnemonic Name	Species	Common Name	Accession Number
DANRE	*Danio rerio*	Zebrafish	A3KNW0
XENLA	*Xenopus laevis*	African clawed frog	A1L1C2
XENTR	*Xenopus tropicalis*	Western clawed frog	Q28DT3
CRIGR	*Cricetulus griseus*	Chinese hamster	A0A3L7GMU4
MOUSE	*Mus musculus*	Mouse	Q5SWZ9
BOVINE	*Bos taurus*	Bovine	NP_001258919.1
BOVINE	*Bos taurus*	Bovine	E1BE10
PANTR	*Pan troglodytes*	Chimpanzee	G2HE65
HUMAN	*Homo sapiens*	Human	Q8N2A8
PONAB	*Pongo abelii*	Sumatran orangutan	A0A2J8RII7
PONAB	*Cercocebus atys*	Sooty mangabey	A0A2K5LCM4

**Table 3:** KEGG bioaccumulation analysis of PLD6.

Potential Biomarker	Proteins Involved (%)	KEGG Pathway ID	Description
PLD6	61.08	map 00564	Glycerophospholipid metabolism
	14.77	map 00440	Aminophosphonate metabolism
	9.55	map 00260	Glycine, serine and threonine metabolism
	7.03	map 00565	Ether lipid metabolism
	7.03	map 04912	GnRH signaling pathway
	0.18	map 00623	2,4-Dichlorobenzoate degradation
	0.18	map 00960	Alkaloid biosynthesis II
	0.18	map 00650	Butanoate metabolism

**Table 4:** Primers used for qRT-PCR.

Gene	Primer Sequence (5′→3′)	Fragment Size (bp)	Gene ID	Accession Number
*GAPDH*	F: TGACCCCTTCATTGACCTTC	250	281181	NM_001034034
	R: TACTCAGCACCAGCATCACC			
*VASA*	F: TTGGGACTTGTGTAAGAGCTGT	98	493725	NM_001278643
	R: CTTGACTTGTTTGAGGC			
*UCHL1*	F: CCTTCCCTGGGCAGGTGCGCGC	280	514394	NM_001046172
	R: GGCTGTAGAACGCAAGAA			
*PLD6*	F: GTGGACAGGAAGGTGCTGAT	108	526651	NM_001271990
	R: TACTCCTCGTCCTCCACGAT			

## Data Availability

The data that support the findings of this study are available from the corresponding author, upon reasonable request.
